# Stem-like TCF1^+^CD8^+^ T cells are associated with improved survival in male glioblastoma patients

**DOI:** 10.1093/noajnl/vdag203

**Published:** 2026-08-03

**Authors:** Juyeun Lee, Jesper Dupont Ewald, Rikke H Dahlrot, Arnon Møldrup Knudsen, Justin D Lathia, Bjarne Winther Kristensen

**Affiliations:** Department of Cancer Sciences, Cleveland Clinic, Cleveland (J.L., J.D.L.); Florida Research and Innovation Center, Cleveland Clinic, Port St. Lucie; Department of Pathology, Odense University Hospital, Odense; Department of Clinical Research, University of Southern Denmark, Odense (R.H.D.); Department of Oncology, Odense University Hospital, Odense; Department of Pathology, Aarhus University Hospital, Aarhus; Department of Clinical Medicine, Aarhus University, Aarhus; Department of Cancer Sciences, Cleveland Clinic, Cleveland (J.L., J.D.L.); Rose Ella Burkhardt Brain Tumor Center, Cleveland Clinic, Cleveland; Case Comprehensive Cancer Center, Cleveland (J.D.L.); Department of Molecular Medicine, Cleveland Clinic Lerner College of Medicine, Case Western Reserve University, Cleveland; Department of Clinical Medicine and Biotech Research and Innovation Centre (BRIC), University of Copenhagen, Copenhagen; Department of Clinical Medicine and Biotech Research and Innovation Centre (BRIC), University of Copenhagen, Copenhagen; The Bartholin Institute, Department of Pathology, Rigshospitalet, Copenhagen University Hospital, Copenhagen

**Keywords:** glioblastoma, sex differences, stem-like CD8+ T cells

## Abstract

Glioblastoma (GBM) exhibits a significant male bias, characterized by higher incidence and worse clinical outcomes. Tumor-intrinsic and extrinsic factors, including the immune system, contribute to these differences, yet the precise mechanisms and clinical implications remain elusive. We previously identified an increased frequency of stem-like/progenitor-exhausted TCF1^+^CD8^+^ T cells in male GBM mouse models and human patients, which may contribute to these sex differences. In this study, we sought to determine the clinical significance of stem-like TCF1^+^CD8^+^ T cells in GBM and their potential to influence survival outcomes in a sex-dependent manner.

T cell factor 1 (TCF1) is a key transcription factor that governs T cell memory formation and maintenance by supporting cellular self-renewal.^1^ TCF1 also serves as a defining marker of progenitor-exhausted T cells, a subset of CD8^+^ T cells that arise during chronic antigen exposure, such as persistent viral infection or tumors.^1^ In the tumor microenvironment, progenitor-exhausted TCF1^+^CD8^+^ T cells play a critical role in sustaining the antitumor immune response by self-renewing and giving rise to more differentiated, effector-like progeny, which eventually become terminally exhausted and lose effector function.^2^ While a higher frequency of progenitor-exhausted TCF1^+^CD8^+^ T cells in males has been reported across multiple cancers,^3^,^4^ the sex-specific clinical outcome association of this population has not been investigated.

To assess the correlation between CD8/TCF1 co-expression and survival in IDH wild-type GBM patients, we analyzed a cohort of 184 newly diagnosed patients (107 males, 77 females) after staining for CD8 and TCF1. The co-expression fraction was defined as the count of CD8-positive cells expressing TCF1 divided by the total count of CD8-positive cells. Analysis revealed that the co-expression fraction ranged from 0% to 18.15% (mean: 4.17%) in males and from 0% to 14.32% (mean: 3.41%) in females. Although males exhibited a slightly higher mean value in co-expression fraction, the difference between the sexes was not statistically significant (*P *= .12).

Survival analysis was performed by stratifying patients into “high” and “low” groups based on either the median or the third-quartile (top 25% vs bottom 75%) of CD8/TCF1 co-expression. In the total cohort, univariate analysis showed no significant differences when using the median cutoff (Figure 1A). However, using the third quartile cutoff, the “high” group demonstrated significantly longer survival compared to the “low” group (HR 0.68, 95% CI, 0.50-0.93, *P *= .0234) (Figure 1A), suggesting a potential threshold effect of TCF1^+^CD8^+^ T cells in driving a survival benefit, particularly within the immunologically cold GBM microenvironment. This association remained significant in a multivariate model (HR 0.89, 95% CI, 0.79-1.0, *P *= .045), accounting for age at diagnosis, sex, and treatment regimen (curative-intended, palliative, or surgical treatment only).

**Figure 1.**
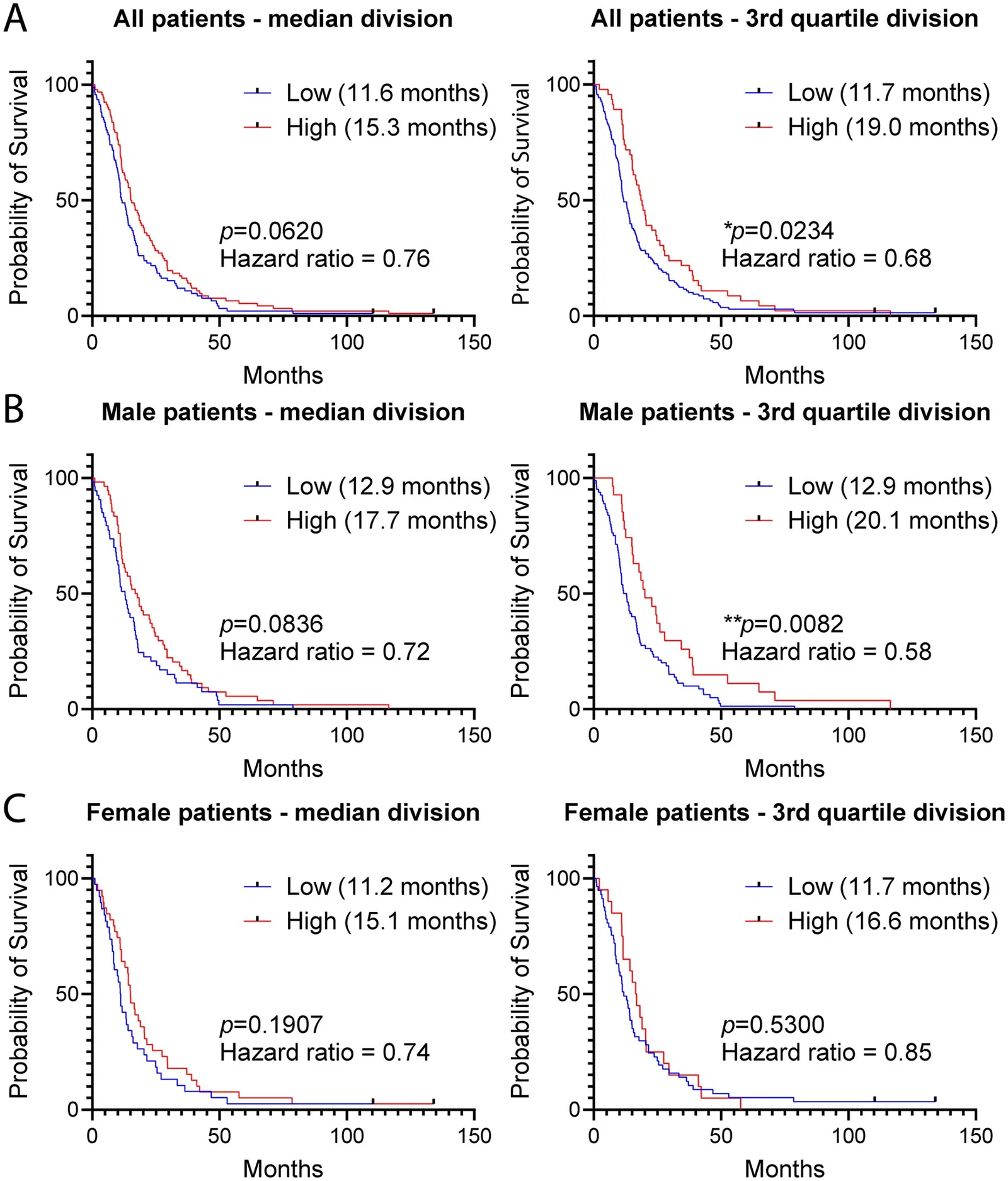
Survival analysis of GBM patients stratified by median or third-quartile division of CD8 and TCF1 co-expression in tissue microarray samples. (A) All patients, (B) Male patients only, (C) Female patients only.

To evaluate the sex-specific effect of TCF1^+^CD8^+^ T cells, we analyzed males and females independently. In males, the median split showed a trend toward increased median survival that did not reach significance (Figure 1B). However, the third quartile split revealed a significant survival advantage for the “high” group (HR 0.58, 95% CI, 0.38-0.84, *P *= .0082) (Figure 1B), further confirmed by multivariate analysis (HR 0.85, 95% CI, 0.73-0.99, *P *= .0031). In contrast, no significant survival differences were observed in females regardless of the stratification method or analysis type (Figure 1C). These results indicate that the positive correlation between the TCF1^+^CD8^+^ T-cell fraction and survival observed in the total cohort was driven primarily by the male population.

TCF1^+^CD8^+^ T cells function as a reservoir that maintains T cell immunity within the tumor microenvironment.^1^ Male patients with low CD8^+^/TCF1 co-expression may have progressed toward a predominantly terminally exhausted T cell state, consistent with impaired tumor control, whereas a higher TCF1^+^ fraction likely reflects a robust progenitor reservoir capable of sustaining antitumor immune responses. The observation that a survival extension is evident only in the third quartile analysis may reflect the degree of tumor-mediated immunosuppression, suggesting that TCF1^+^CD8^+^ T cells may serve as a potential biomarker in males. Notably, this prognostic association is absent in females, suggesting that GBM survival in females may be driven by mechanisms distinct from the TCF1^+^CD8^+^ T cell compartment.

A limitation of this study is that TCF1^+^CD8^+^ T cells were defined based on TCF1 expression alone, without additional markers to distinguish naïve T cells from stem-like memory or progenitor-exhausted subsets. In GBM, the majority of tumor-infiltrating CD8^+^ T cells are antigen-experienced rather than naïve,^5^ making it likely that TCF1^+^CD8^+^ T cells in this context predominantly represent stem-like memory and/or progenitor-exhausted subsets. Both populations are characterized by self-renewal capacity and the ability to sustain more differentiated effector populations, consistent with our focus on global stem-like TCF1^+^CD8^+^ T cells within the tumor microenvironment. Future studies incorporating a broader immunophenotyping panel, such as CCR7, PD-1, and TIM-3, will be needed to more precisely define the T-cell state driving the observed survival association.

In addition, TCF1^+^CD8^+^ T cells are known to give rise to effector-like populations following PD-1-targeted immunotherapy,^6^ and male patients have demonstrated greater responsiveness to immune checkpoint blockade across multiple cancer types.^7^ Consistent with our previous observations in a mouse GBM model, where anti-PD-1 treatment conferred benefit only in males,^3^ our data suggest that TCF1^+^CD8^+^ T cells may mediate sex differences in response to immunotherapy and highlight the importance of incorporating biological sex as a key variable in future clinical trials. Future studies will focus on elucidating why the TCF1 reservoir is uniquely predictive in males, with an emphasis on epigenetic regulation mediated by TCF1, which could inform the development of sex-stratified immunotherapeutic strategies.

## Data Availability

Data can be made available upon request to the corresponding author, Bjarne Winther Kristensen (bjarne.winther.kristensen.01@regionh.dk).
